# Colorimetric detection of chloroperoxyl radical in reactive chlorine species solutions

**DOI:** 10.1371/journal.pone.0334046

**Published:** 2025-10-21

**Authors:** Hiroyuki Kawata, Shunsuke Odai, Hisataka Goda, Yuki Okawa, Toshiaki Kamachi

**Affiliations:** 1 Department of Life Science and Technology, Institute of Science Tokyo, Ookayama, Meguro-ku, Tokyo, Japan; 2 Sankei Co. Ltd., Shiromi, Chuou-Ku, Osaka, Japan; National Research and Innovation Agency, INDONESIA

## Abstract

Chlorous acid water (CAW) is a chlorine-based disinfectant approved as a food additive in Japan. CAW is synthesized by the reaction of chloric acid aqueous solution with hydrogen peroxide under acidic conditions. However, in this synthesis method, various ions**—**such as Na^+^ from NaClO_3_ and the conjugate base of the acid used**—**remain in the solution, hindering the selective detection and decreasing stability of chloroperoxyl radical (ClOO•), a potential key disinfectant species. In this study, we aimed to establish a colorimetric quantification method for ClOO•. We prepared a high-purity ClOO• solution (ClO_2_cx_) by cation exchange and its purity and stability were evaluated using electron spin resonance (ESR) spectroscopy and total chlorine concentration measurements. Furthermore, several colorimetric methods—including DPD, TMB, and DPPH assays—were examined to quantify ClOO•, and their sensitivity and selectivity were comparatively assessed. ClOO• was the sole detectable oxidant in the solution, with a lifetime exceeding 100 h, indicating exceptional stability under ambient conditions. Among the evaluated colorimetric methods, the DPD-based method was found to be suitable for quantifying ClOO•, showing a wide detection range and excellent linearity. This study represents the first report of a colorimetric quantification method for ClOO•. Our findings are expected to be useful for quantitatively discussing the biological efficacy of ClOO• and its reaction mechanisms.

## Introduction

Chlorine-based disinfectants, such as hypochlorous acid solution and chlorine dioxide gas, exert a potent sterilizing effect by denaturing proteins and disrupting the structural integrity of microbial cells and viral particles [1,2]. These agents are particularly effective against a wide range of pathogens, including alcohol-resistant microorganisms and viruses such as non-enveloped viruses and bacterial spores. Their ability to achieve high antimicrobial efficacy at relatively low concentrations also minimizes toxicity and material degradation [3–5]. Due to their broad-spectrum effectiveness and versatility across healthcare, food safety, and public hygiene settings, the demand for chlorine-based disinfectants as an alternative to alcohol-based ones is increasing annually [6–12].

Among chlorine-based disinfectants, particular attention has been given to chlorous acid water (CAW) and acidified sodium chlorite (ASC). CAW, approved as a food additive in Japan, is produced through a multi-step process involving electrolysis and reduction reactions [13]. In contrast, ASC is authorized in the United States for use as an antimicrobial agent in food processing and can be prepared by mixing sodium chlorite with Generally Recognized As Safe (GRAS) acids [14]. Our previous studies have revealed that the chloroperoxyl radical (ClOO•) is the predominant active species in ASC. Despite their effectiveness, however, both CAW and ASC face challenges in long-term storage due to residual reactants etc [15,16].

Residual reactants and by-products may interfere with the accurate evaluation of ASC and CAW, particularly in the quantification of ClOO•. Disinfectant performance is typically assessed using oxidation-reduction potential (ORP) measurements or potassium iodide (KI) colorimetric methods as indicators of oxidative strength [17–19]. However, these methods also account for species with no or low antimicrobial activity, making them unsuitable for accurately reflecting the actual performance of ASC and CAW. Therefore, selective quantification of ClOO• is essential. Currently, electron spin resonance (ESR) spectroscopy is the only established method for this purpose [20]. However, its limited sensitivity and lack of instrument versatility pose significant challenges. Therefore, the development of a simpler and more sensitive quantification method is strongly needed.

To address this challenge, we focused on colorimetric quantification methods. Such methods do not require complex instrumentation, allow rapid and simple evaluation of specific species, and can potentially achieve both selectivity and sensitivity through optimization of reaction conditions. In fact, colorimetric methods using reagents such as DPD and TMB are widely employed for quantifying oxidants, including chlorine-based disinfectants, and may represent a promising approach for ClOO• determination.

**Scheme 1:**
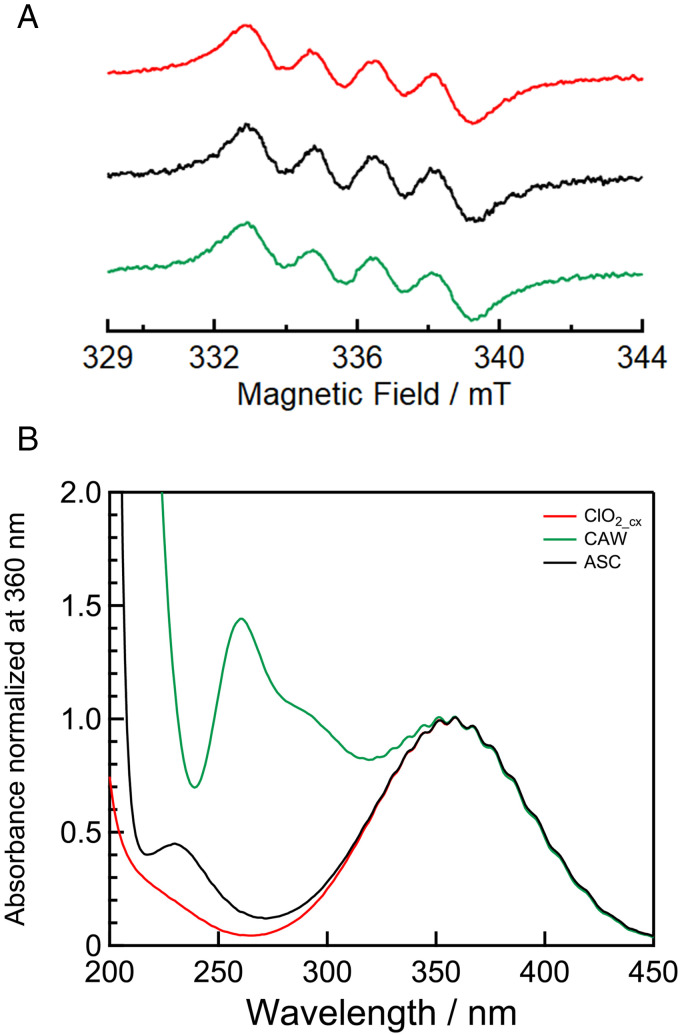
Synthesis pathway for HClO_2_ in CAW, ASC and ClO_2_cx_.

In this study, we aimed to establish a colorimetric method for quantifying ClOO• by evaluating the applicability of chromogenic reagents. To begin, we prepared a high-purity chlorous acid solution (ClO_2_cx_) via cation exchange of a sodium chlorite aqueous solution (Scheme 1). The purity and temporal stability of ClOO• in ClO_2_cx_ were then evaluated using ESR spectroscopy. Finally, we propose a novel colorimetric method that enables simple and highly sensitive quantification of ClOO•, which has until now been evaluated only by ESR.

## Methods

### Chemicals

Sodium chlorite (purity: 80%) was purchased from FUJIFILM Wako Pure Chemical Co., Ltd. Chlorine dioxide solution (ClO_2_(GB-standard)) that conforms to the Chinese Hygienic Standard (GB 26366−2010) was purchased from Dongying Shengya Chemical Co., Ltd. CAW was purchased from Honbu Sankei Co., Ltd. All other chemicals were of analytical grade and purchased commercially without further purification unless otherwise specified.

### Preparation of ClO_2_cx_

Cationic exchanged ClO_2_(GB-standard), abbreviated as ClO_2_cx_, was prepared according to the following process [21]: Briefly, to diluted ClO_2_(GB-standard) was added an equal amount of Amberlite IR120B(H)-HG resin, and the mixture was stirred for 24 h in a sealed bottle at 4 °C. After completion of the cation-exchange reaction, the supernatant (ClO_2_cx_) was transferred to sealed PET bottles and stored in a refrigerator.

### Preparation of reactive chlorine species (RCS) solutions

As a comparison of ClO_2_cx_, the following three types of RCS solution, which can be prepared in the laboratory, were used in this study.

Acidified sodium chlorite (ASC) was prepared by mixing equal volume of 6.25–100 mM NaClO_2_ solution and 2.5N HCl [20]. The mixture was allowed to react for 240 s and used immediately thereafter.

Chlorine dioxide solution (CDS) was prepared according to the following procedures using the reaction system shown in S1 Fig [22]. To 750 mL of 0.15 M NaClO_2_ aq. was added 20 mL of sulfuric acid (98% (v/v)) with stirring in a three-necked round-bottom flask. The generated chlorine dioxide gas was transferred through silicon tube and bubbled into RO water by introducing N_2_ gas into the flask at a flow rate of 110 ± 10 mL/min. The reaction was stopped after 120 min, when the FAC level (described below) of CDS reached its maximum, and the resulting solution was stored at −80 °C.

Chlorine solution was prepared according to the following procedures using the same reaction system as CDS [23]. To 300 g of 5% (w/v) NaClO aq. was added 100 g of 20% H_2_SO_4_ aq. in a three-necked round-bottom flask. The generated chlorine gas was transferred through silicon tube and bubbled into RO water by introducing N_2_ gas into the flask at a flow rate of 110 ± 10 mL/min for 30 min to obtain chlorine solution.

The total available chlorine (TAC) and free available chlorine (FAC) levels of RCS solutions were determined by iodometric titration method and DPD method, respectively [24]. Detailed methods are mentioned in the supporting information.

### ESR measurement [20]

A sample solution was placed into a glass capillary tube 120 mm long with a 0.8 mm inner diameter. The glass capillary was transferred into a capillary cell fitted in an ESR cavity, and ESR measurements were carried out at room temperature using an X-band spectrometer (JEOL FA-100ESR) operated at 9.46 GHz; the magnetic field was modulated at 100 kHz. The conditions for measuring the ESR spectra were as follows: resonant frequency = 9.46 GHz, microwave power = 4 mW, observed magnetic field = 336.0 ± 7.5 mT, field modulation width = 0.1 mT, sweep time = 4 min, and time constant = 0.1 s. The concentrations of radicals were determined using TEMPOL as an external standard. Radical species were quantified using integration software to determine the spectrum area mounted in the ESR device.

### Stability test for ClOO•

A 100 mL aliquot of each ClOO•-containing solution (ClO_2_cx_, CDS, and ASC) was transferred into individual beakers and left unsealed in a fume hood at room temperature under ambient lighting. At designated time points, 1 mL aliquots were collected for concentration measurement using ESR at the indicated time. The decay of ClOO• concentration over time was fitted to a single exponential curve, and the corresponding lifetimes were estimated.

### Determination of ClOO• concentration by spectrophotometry

The concentration of ClOO• in ClO_2_cx_ was determined by DPD method [24]. Briefly, the test sample (9.5 mL) was buffered with 0.5 mL of 0.2 M KH_2_PO_4_ (pH6.5), after which 0.1 g of DPD-Na_2_SO_4_ mixture (1:24 w/w, ground) was added. The absorbance at 510 nm was then measured.

## Results

### Radical species in ClO_2_cx_

**Fig 1:**
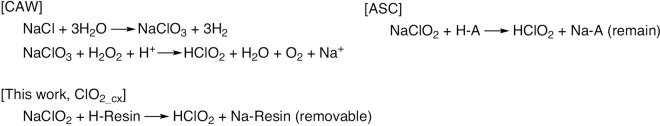
ESR spectra (a) and UV-Vis absorption spectra (b) of ClO_2_cx_ (red), ASC (black) and CAW (green). Absorption spectra were normalized at 360 nm.

The radical species of ClO_2_cx_, which is prepared by acidifying ClO_2_(GB-standard) by cation exchange, were directly detected by ESR spectroscopy. As shown in Fig 1a, ClO_2_cx_ exhibited an ESR signal at room temperature, and the signal was split into four lines with an intensity ratio of 1:1:1:1, similar to that of ASC. Analysis of ESR spectrum led to the hyperfine splitting constant of 1.70 mT and g-value of 2.0125. These results indicate that the radical active species in ClO_2_cx_ is the chloroperoxyl radical (ClOO•) reported in our previous work [20].

We compared the concentration of RCSs (TAC level) in samples determined by the iodine titration method. For ClO_2_cx_, the ClOO• concentration determined from ESR matched that from iodine titration, indicating ClOO• as the main component of RCS. In contrast, for ASC, TAC level was 1.14 times higher than ClOO• concentration, suggesting the presence of other active species. These findings indicate that acidifying ClO_2_(GB-standard) using a cation-exchange resin is a more effective method for preparing ClOO• than adding an excess amount of acid such as hydrochloric acid or sulfuric acid. This is likely due to the stoichiometric exchange of sodium ions in ClO_2_(GB-standard) for protons by the cation exchange resin.

To identify reactive species other than ClOO•, UV-Vis absorption spectra of ClO_2_cx_, ASC, and CAW were measured. As shown in Fig 1b, ClO_2_cx_ exhibited a characteristic absorption band with a maximum absorption at 365 nm at room temperature, which was suggested to be derived from ClOO• [20]. This band was also observed in ASC and CAW, however, CAW uniquely displayed an additional absorption band at 260 nm and a shoulder band at around 290 nm, which are attributed to chlorite ions [25]. ASC also displayed an additional band at 230 nm, which is attributed to hypochlorous acid [26]. These results suggest that CAW and ASC contain not only ClOO• but also RCSs.

### Stability of chloroperoxyl radical

**Fig 2:**
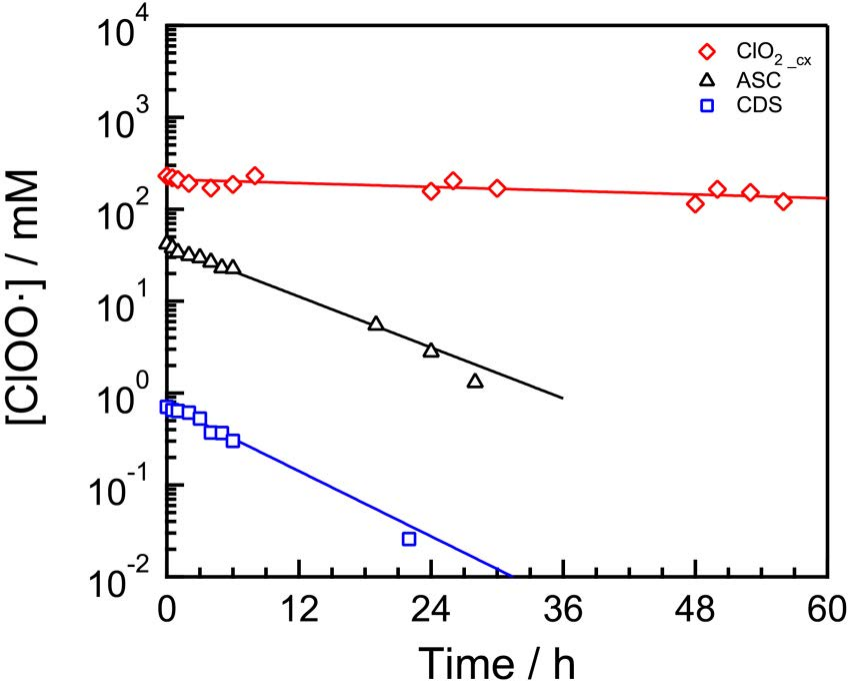
ClOO• stability of ClO_2_cx_, CDS or ASC. The sample solutions were left unsealed in the hood under ambient light conditions at room temperature. ClOO• concentration was determined by ESR measurement. The decay data were fitted to single-exponential curve. The lifetimes of ClOO• were 7.3 h for CDS, 9.4 h for ASC, and 130 h for ClO_2_cx_.

Radicals such as hydroxyl radicals have a very short lifetime due to their high reactivity, making them difficult to directly detect using ESR. On the other hand, ClOO• was stable enough to be directly detected by ESR, so we evaluated its stability in several solutions containing ClOO• (Fig 2). In the case of ASC and CDS, only 10% or less of the initial concentration of ClOO• remained after 24 h, whereas 70% remained in ClO_2_cx_. The lifetimes of ClOO• were calculated to be 7.3 h for CDS, 9.4 h for ASC, and 130 h for ClO_2_cx_ as estimated by single-exponential curve fitting of the decay curve. These results suggest that ClO_2_cx_ has an extremely long lifetime compared to other ClOO•-containing solutions and is suitable for long-term storage.

### ClOO• quantification by colorimetric method

The concentration of ClOO• can be determined by ESR measurement, but it has low detection sensitivity and requires large-scale equipment, necessitating a more conventional method. As shown in Fig 1b, ClOO• exhibits absorption around 365 nm, allowing for concentration calculation from absorbance. However, the lower limit of quantification is nearly the same as that of ESR measurement.

**Fig 3:**
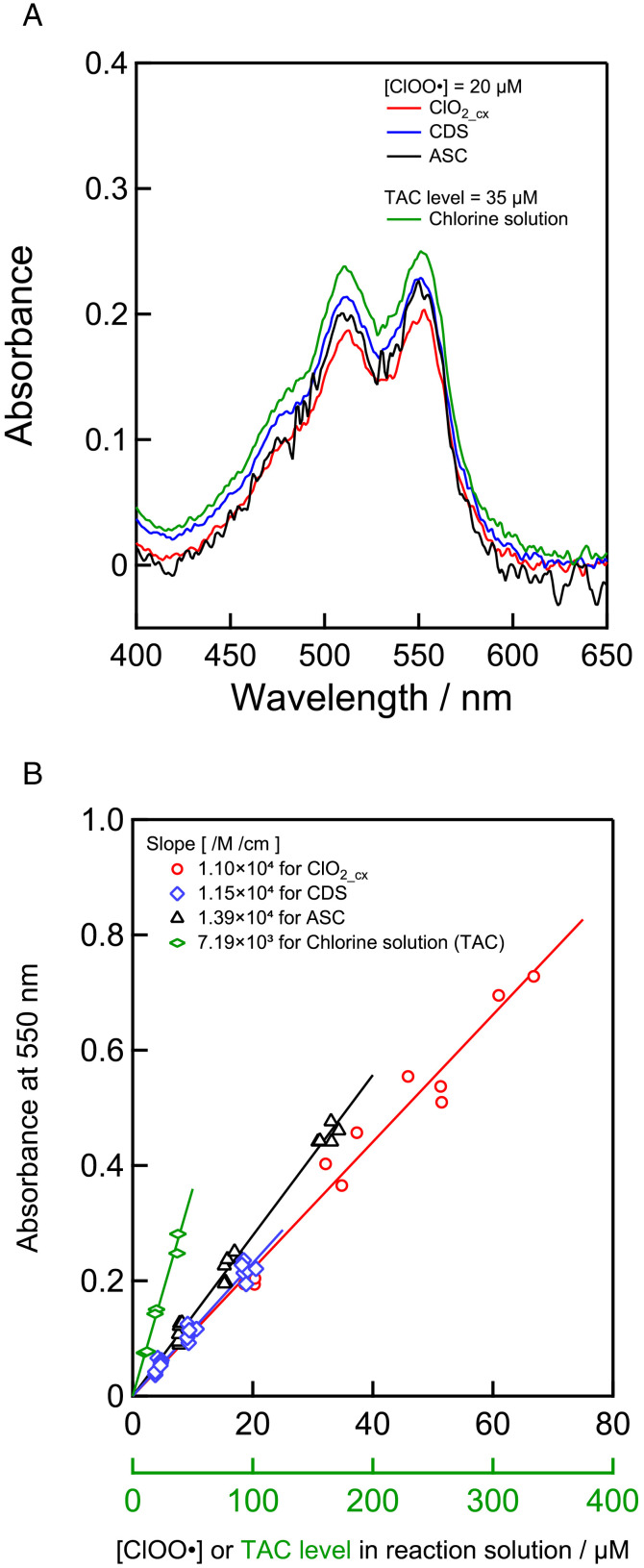
ClOO• determination using DPD method. (a) Absorption of DPD reaction solution with 20 μM ClOO•-containing solution (red, ClO_2_cx_; blue, CDS; black, ASC) and chlorin solution (green, TAC level = 35 μM as Na_2_S_2_O_3_). (b) Calibration curves of DPD method for ClOO• in different RCS solutions at 550 nm.

In this study, colorimetric methods for RCS detection (DPD, DPPH or TMB methods) were evaluated for measuring ClOO• concentration [27–29]. DPD and TMB react with oxidants to produce their corresponding oxidized forms with a maximum molar extinction coefficient of 10^4^ M⁻^1^ cm⁻^1^ or more [30,31], allowing for highly sensitive detection of oxidants. DPPH, a stable radical with a maximum molar extinction coefficient of 10^4^ M⁻^1^ cm⁻^1^ or more [4], reacts with antioxidants and other radicals to be quenched, allowing for the highly sensitive detection of quenchers. In addition to examining the detection method, we evaluated the use of chlorin solution, CDS, and ASC, which can be prepared in the laboratory, as standard solutions for quantitative analysis. As shown in Fig 3, the change in absorbance was linear with respect to the ClOO• concentration or TAC level in all solutions using the DPD method. The slope of the plot was almost identical for all solutions containing ClOO•, but it differed for the chlorine solution. These results suggest that it is possible to measure the concentration of active species in chlorous acid solutions using a DPD method with a solution containing ClOO•, especially CDS, as a standard.

## Discussion

ClO_2_cx_ was prepared by treating sodium chlorite solution with a cation exchange resin and exhibited ESR and UV-Vis absorption spectra similar to those of ASC, suggesting the presence of ClOO• as the dominant RCS. This was further confirmed by combining total active species quantification using the KI method with ESR analysis, which demonstrated that ClOO• was the only detectable active species in the solution. The electrochemical synthesis method developed by Zhao et al., which uses a Ti₄O₇ anode, is highly efficient and selective for generating high-purity ClO_2_, both in gaseous form and as a dissolved solution (that is, a ClOO•-containing solution) [32]. While this method requires specialized electrodes, our developed ClO_2_cx_ employs a readily available cation-exchange resin, making it a more convenient and practical alternative for producing pure ClOO•-containing solutions.

The stability of ClOO• was evaluated by incubating three distinct radical-containing solutions over several days. ClOO• exhibited marked instability in ASC and CDS; however, in ClO_2_cx_, it demonstrated a lifetime of approximately 130 h, indicating exceptional stability for a radical species. This observed lifetime substantially exceeds that of ClO_2_ dissolved solution stored at room temperature in a sealed container, as reported by Karadirek et al [33]. In contrast, Kim et al. reported that using organic acid solutions adjusted to specific pH values enables the generation of ClO_2_ with a concentration that remains for at least 14 days [16]. This extended stability was attributed to a slower generation rate of ClO_2_. However, it is important to note that the findings reported in both studies were based solely on ClO_2_ measurements and did not involve the detection or analysis of ClOO• radical. In our study, we demonstrated an unprecedented level of stability for ClOO•, a radical species present in ClO_2_cx_, highlighting its remarkable longevity—especially considering its radical nature. The extended stability of ClOO• in ClO_2_cx_ is attributed to the specific composition and physicochemical conditions of the solution. As shown in S1 Table, TAC level in ASC exceeds the ClOO• concentration, suggesting the presence of other RCS, such as hypochlorous acid, which may facilitate reaction and accelerate ClOO• decomposition [34]. Furthermore, the higher pH of CDS relative to ClO_2_cx_ enhances redox reactions involving ClOO• itself, thereby contributing to its reduced stability [35]. In contrast, ClO_2_cx_ is presumed to contain highly pure ClOO• and to maintain an acidic environment, both of which are considered key factors in its enhanced stability.

Investigation into method for measuring the concentration of ClOO• revealed that the DPD is the most suitable indicator. In all ClOO•-containing solutions, the absorbance of DPD exhibited good linearity with ClOO• concentration. The slope of plot was calculated to be 1.1 × 10^4^ M^−1^ cm^−1^ per ClOO•, which is exactly half that reported by Bader et al. and Zhu et al. (2.1 × 10^4^ M^−1^ cm^−1^) [31,36], where DPD was oxidized by H_2_O_2_ or IO₄⁻ at a 2:1 molar ratio. This suggests that DPD reacts with ClOO• in a 1:1 molar ratio, indicating that the chemical species generated by the one-electron reduction of ClOO• does not further react with DPD. In contrast, the TMB method, which detects active species *via* a similar mechanism to DPD method [4], showed a narrower linear range for ClOO• detection, indicating a lower dynamic range. Moreover, the TMB method exhibited a more pronounced difference in responsiveness between ASC and CDS, and was more susceptible to interference from byproducts. In the DPPH method, an increase in absorbance was observed, contrary to the typical decrease seen in antioxidant assay [37]. The absorption spectra varied in shape depending on the sample, indicating that the DPPH method is highly sensitive to pH and byproducts [38–40], making it unsuitable for accurate ClOO• quantification. Few studies have reported the reaction of DPPH with ClOO• or HClO_2_, and the mechanism of these reactions remain unclear, within the scope of our investigation. In the presence of strong oxidants, a complex radical chain reaction proceeds, and it is believed that ClOO• participates in these processes. Based on these results, we report for the first time that the DPD method enables reliable and highly sensitive quantification of ClOO•, which had previously been measurable only using ESR. In the future, this method is expected to be effective for discussing the disinfectant efficacy of ClO_2_cx_, including the concentration of ClOO•.

## Conclusion

We successfully prepared a solution containing high-purity ClOO•, referred to as ClO_2_cx_, through cation exchange method. This solution is free from any oxidatively active species other than ClOO• and exhibits very high ClOO• stability. We further investigated a method for measuring the concentration of ClOO• and found that ClOO• could be accurately quantified using DPD method. We are currently applying ClO_2_cx_ to study its bactericidal action and investigating its reaction mechanism.

## Supporting information

S1 Fig Schematic diagram of the reaction system for CDS and chlorine solution preparation. (TIF)

S2 Fig UV-Vis absorption spectra of CAW. The measured absorption spectrum of CAW indicated that it is a mixture of ClOO• and chlorite ion, as the spectra are given by the sum of these compounds.
(TIF)

S3 Fig ESR spectra of CDS and chlorine solution. Chlorine solution was measured with Mn marker.
(TIF)

S4 Fig ClOO• determination using TMB (a, b) and DPPH (c,d) method. (a, c) Absorption spectra of the reaction solution with RCS. (b, d) Calibration curves for ClOO•.
(TIF)

S1 Table The purity of ClOO• and pH value of RCS solutions used in this study. TAC level was calculated as ClOO• equivalent by iodometric titration and ClOO• concentration was measured by ESR. pH value was measured using portable pH meter (HORIBA, LAQUAtwin).
(TIF)
