# Supplementary figures and images for: Colorimetric detection of chloroperoxyl radical in reactive chlorine species solutions
Source: PLoS One. 2025 Oct 21;20(10):e0334046. doi: 10.1371/journal.pone.0334046 (PMC12539726; doi:10.1371/journal.pone.0334046)

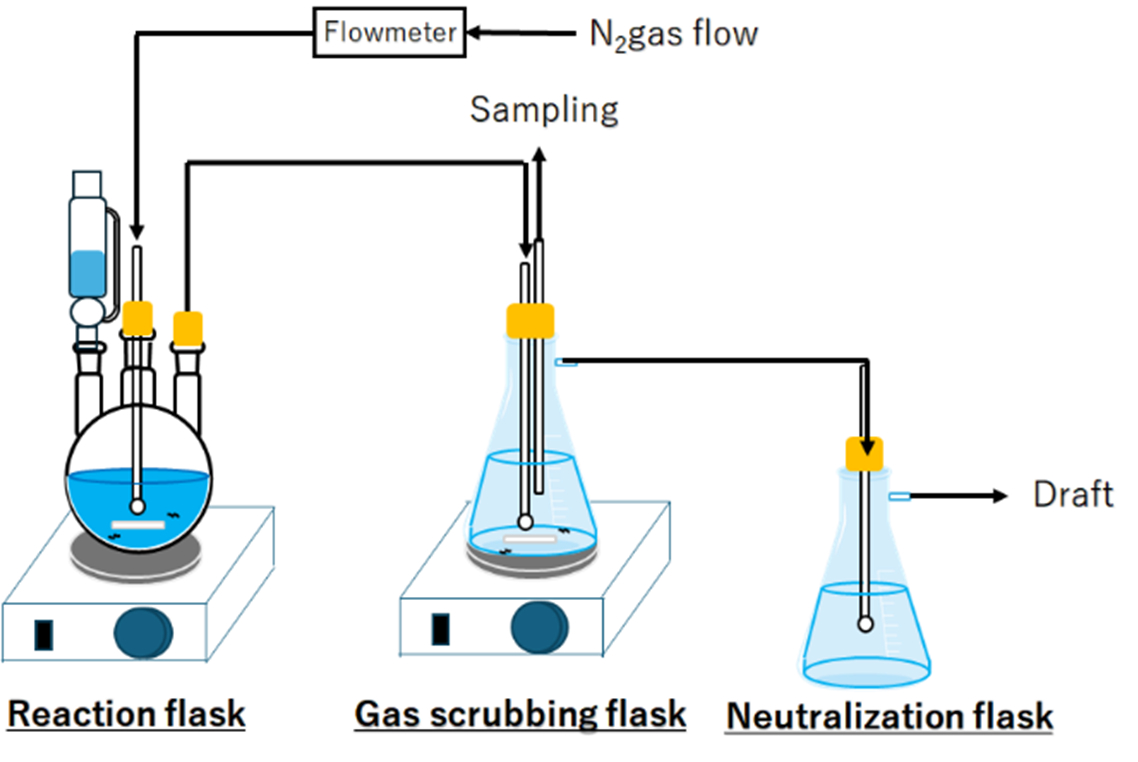

Supplement: S1 Fig — (TIF) [file pone.0334046.s001.tif]

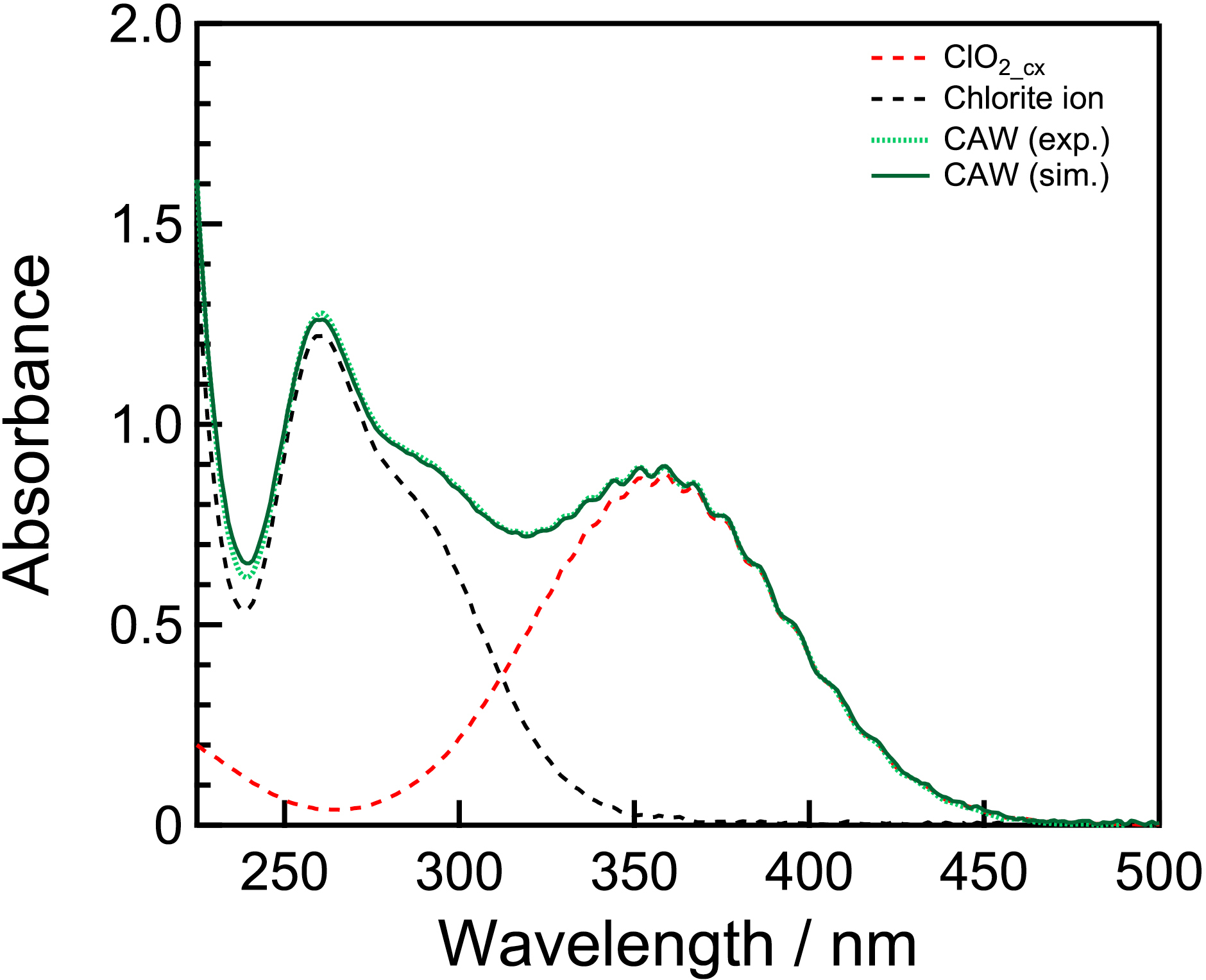

Supplement: S2 Fig — The measured absorption spectrum of CAW indicated that it is a mixture of ClOO• and chlorite ion, as the spectra are given by the sum of these compounds. (TIF) [file pone.0334046.s002.tif]

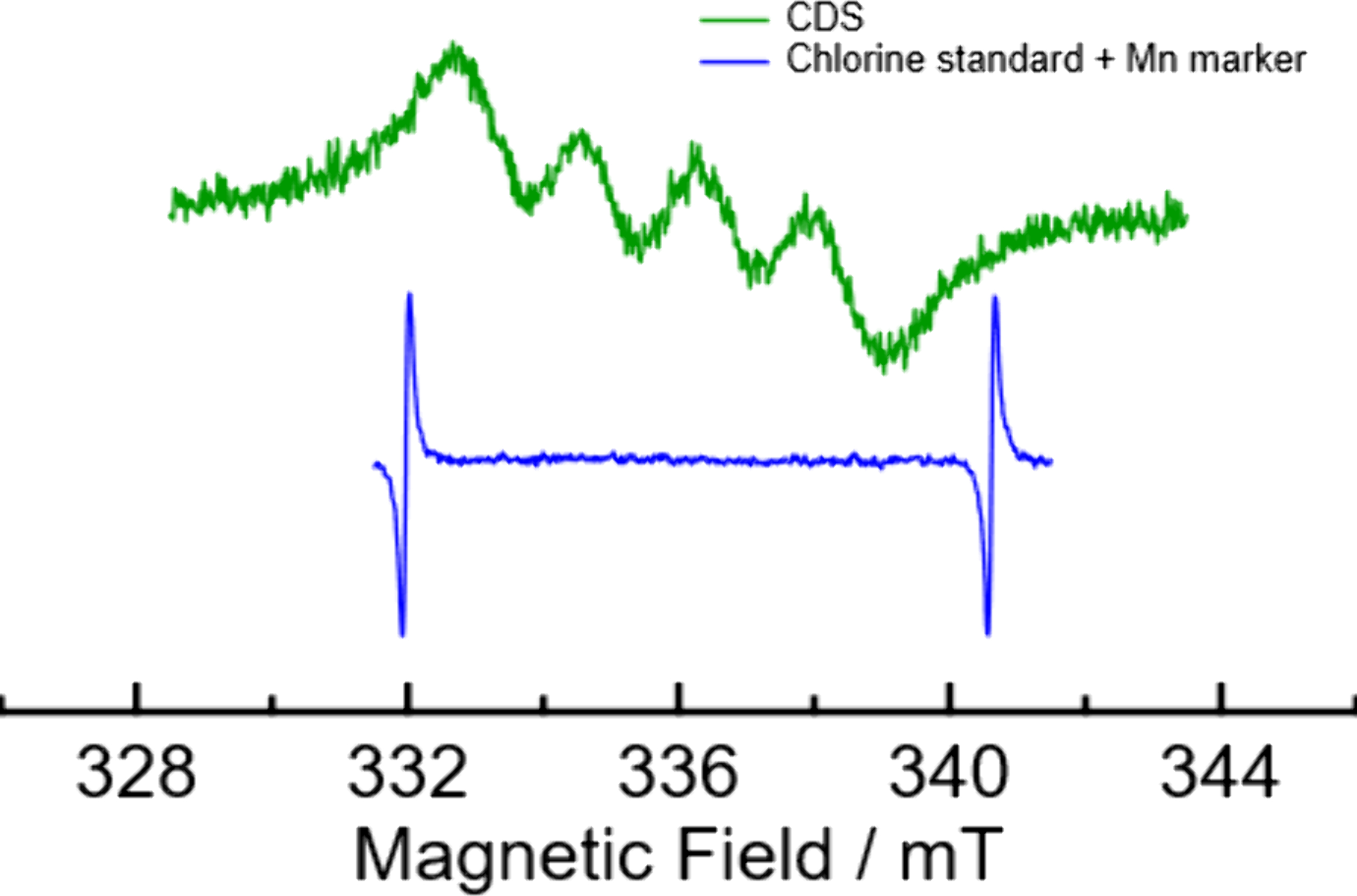

Supplement: S3 Fig — Chlorine solution was measured with Mn marker. (TIF) [file pone.0334046.s003.tif]

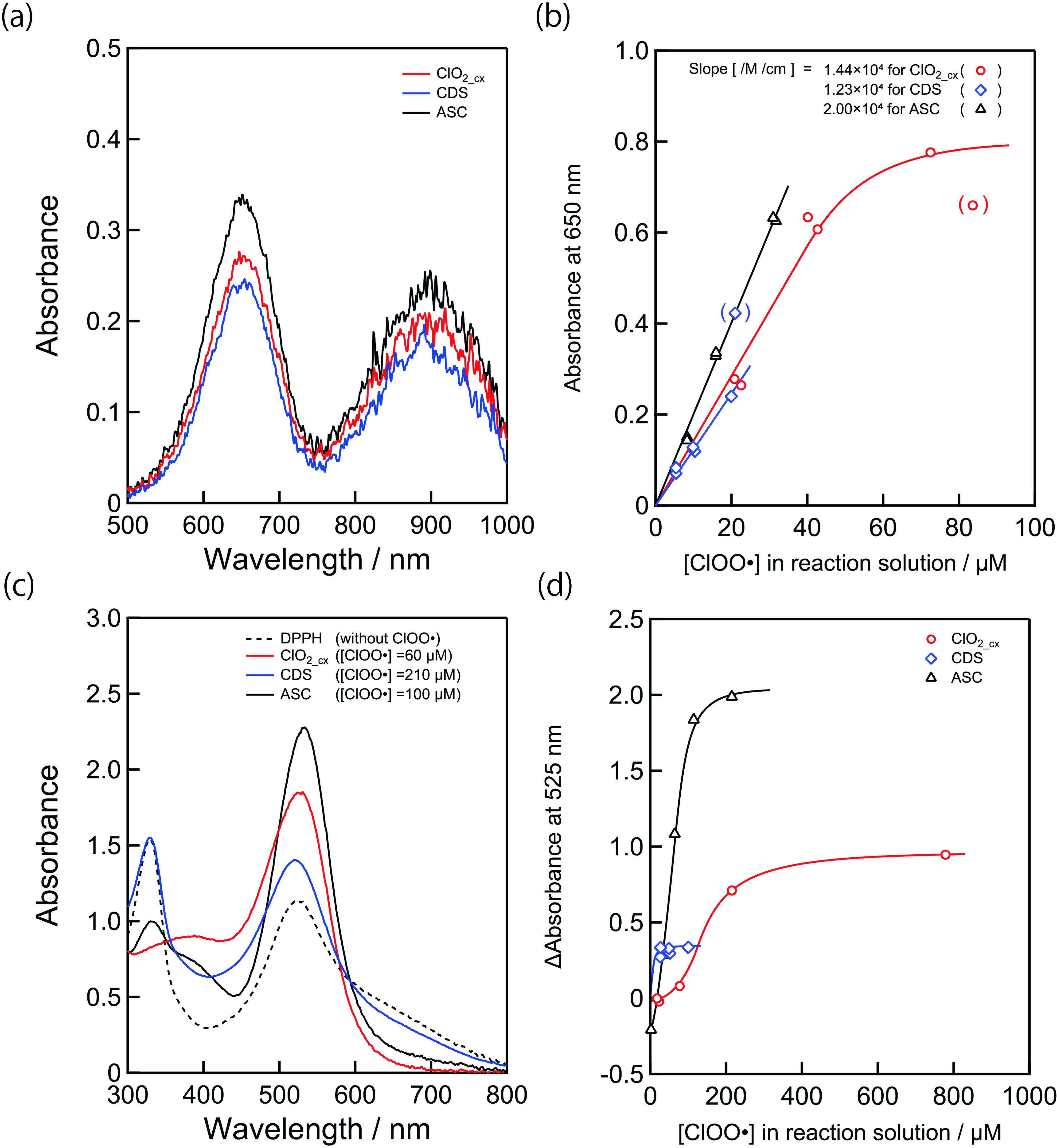

Supplement: S4 Fig — (a, c) Absorption spectra of the reaction solution with RCS. (b, d) Calibration curves for ClOO•. (TIF) [file pone.0334046.s004.tif]

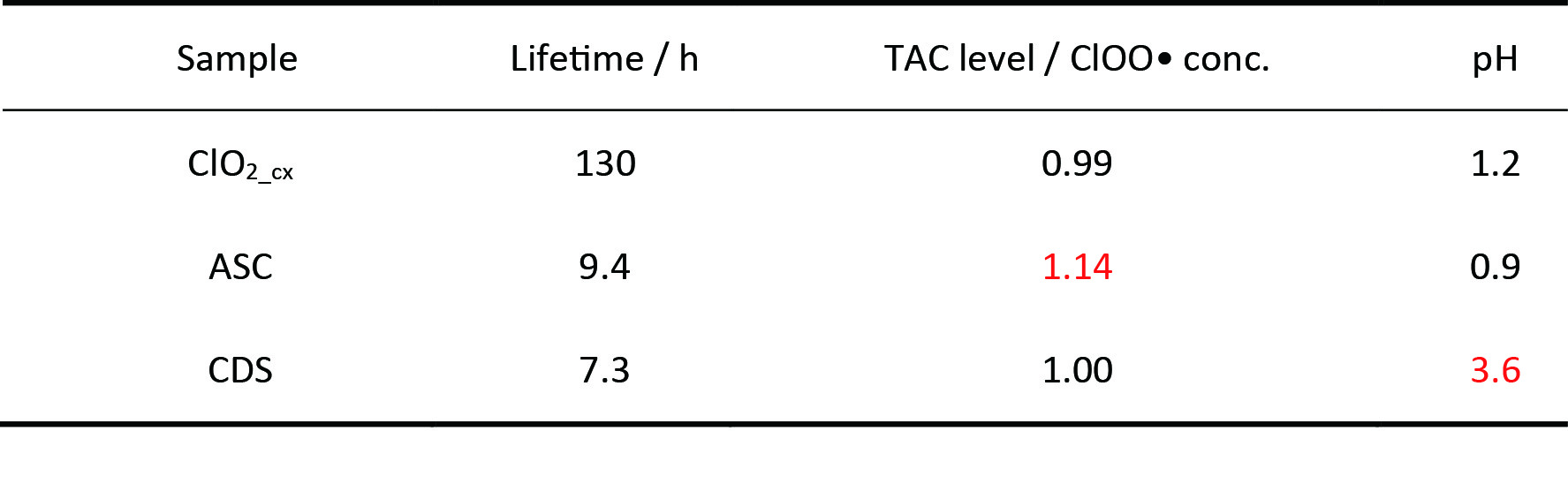

Supplement: S1 Table — TAC level was calculated as ClOO• equivalent by iodometric titration and ClOO• concentration was measured by ESR. pH value was measured using portable pH meter (HORIBA, LAQUAtwin). (TIF) [file pone.0334046.s005.tif]
